# Trends in Liver Cirrhosis and Diabetes-Related Mortality Among Adults in the United States: A CDC WONDER Database Analysis (1999–2020)

**DOI:** 10.3390/life15060852

**Published:** 2025-05-25

**Authors:** Manahil Irfan, Rameesha Ahmad, Mariam Arshad Ahmed, Ayan Mohammed Khan, Zoya Aamir, Rayaan Imran, Raheel Ahmed

**Affiliations:** 1 Medical College, Aga Khan University, Karachi 74800, Pakistan; manahil.irfan@scholar.aku.edu (M.I.); rameesha.ahmad@scholar.aku.edu (R.A.); mariam.ahmed@scholar.aku.edu (M.A.A.); ayan.khan@scholar.aku.edu (A.M.K.); 2 Department of Medicine, Dow Medical College, Dow University of Health Sciences, Karachi 74200, Pakistan; zoyaaamirsid@gmail.com (Z.A.); rayaanimran2002@gmail.com (R.I.); 3 National Heart and Lung Institute, Imperial College London, London SW7 2AZ, UK

**Keywords:** liver cirrhosis, diabetes mellitus, mortality trends, United States CDC WONDER, chronic liver disease

## Abstract

**Background:** The burden of liver cirrhosis correlated with diabetes mellitus (DM) poses a significant public health challenge in the United States. Both conditions independently contribute to high morbidity and mortality rates. While extensive individual analyses have been conducted, US trends in comorbid liver cirrhosis-DM-related mortality remain unexplored. This study seeks to investigate mortality trends associated with the simultaneous occurrence of liver cirrhosis and DM among U.S. adults over the period from 1999 to 2020. **Methods:** We conducted a descriptive analysis using publicly available mortality data from the CDC Wide-Ranging Online Data for Epidemiologic Research (WONDER) database. Age-adjusted mortality rates (AAMRs) per 100,000 individuals were calculated using the 2000 U.S. standard population. Trends were stratified by year, age, sex, race/ethnicity, urbanization, region, and state. Joinpoint regression analysis was employed to determine annual percentage changes (APCs) and assess statistical significance (*p* < 0.05). **Results:** A total of 90,418 deaths were recorded among adults with both cirrhosis and DM between 1999 and 2020. The overall AAMR increased from 1.02/100,000 in 1999 to 1.78/100,000 in 2020, reflecting a significant upward trend in mortality. The highest mortality rates were observed in non-metropolitan regions, in the South, and among males, older adults (65+), and NH American Indian or Alaska Native and Hispanic populations. All demographic groups exhibited a pronounced mortality surge between 2018 and 2020. A state-level analysis revealed notable disparities, with Oklahoma and Texas presenting the highest AAMRs. **Conclusions:** Liver cirrhosis and diabetes-related mortality have been steadily increasing over the past two decades, with notable disparities in demographics and regions. These findings underscore the urgent necessity for targeted prevention, early intervention, and policy-level strategies specifically designed for high-risk populations to reduce future mortality rates in the US and ultimately, globally.

## 1. Introduction

Diabetes is defined as a spectrum of metabolic disorders characterized by persistently elevated blood glucose levels due to inadequate insulin secretion, diminished insulin sensitivity, or both [1]. Globally, diabetes stands as one of the foremost public health concerns, with its incidence expanding rapidly across varied populations. This disease has a well-established association with liver cirrhosis, a long-term liver condition in which repeated injury causes healthy liver cells to be replaced by scar tissue, impairing standard functionality [2], thus naming this connection hepatogenous diabetes [3].

Cirrhosis is presently the 11th leading cause of death worldwide [4], 10th in Africa, 9th in both Southeast Asia and Europe, and 5th in the Eastern Mediterranean [5]. In 2016, U.S. liver-related healthcare costs reached $32.5 billion, driven by hospital expenses rising 4% annually [6]. Diabetes affected 529 million people globally in 2021, with a prevalence of 6.1% [7]. Up to 96% of cirrhosis patients experience glucose intolerance, and 30% develop diabetes [8], with impaired glucose metabolism worsening as cirrhosis advances. Impaired glucose metabolism worsens as liver disease progresses, with 60–80% [9,10] facing glucose intolerance and 7–15% developing diabetes. By 2050, diabetes cases may exceed 1.31 billion, and cirrhosis rates are expected to rise [7].

This study aims to identify U.S. mortality trends from 1999 to 2020 for patients with both diabetes mellitus (DM) and liver cirrhosis. Although the mortality patterns of each condition have been studied individually, the joint mortality impact and corresponding prevention strategies remain unaddressed. This will allow healthcare workers, administration, and policymakers to design effective strategies and interventions and deploy appropriate preventative measures.

We hypothesize that mortality due to coexisting cirrhosis and diabetes has increased significantly over time, with disproportionately higher trends among rural populations, older adults, and racial/ethnic minorities.

## 2. Methods

### 2.1. Study Setting and Population

We conducted a descriptive analysis of cirrhosis-related mortality among individuals from 5 to 85+ years old with diabetes mellitus (DM) from 1999 until 2020. This study used mortality data from the Centers for Disease Control and Prevention’s Wide-Ranging Online Data for Epidemiologic Research database (WONDER), which compiles death certificate records from all 50 states and the District of Columbia. Our primary objective was to examine mortality trends associated with the co-occurrence of liver cirrhosis and DM using the International Statistical Classification of Diseases 10th Revision (ICD-10) diagnostic classifications (Supplementary Table S1). Age groups were selected to exclude infants, thus focusing on adults, with evenly spaced intervals to ensure methodological consistency and relevance in the results. The publicly accessible CDC-WONDER online databases offered anonymized, county-level data on vital statistics, inclusive of information on births, deaths, injury-related mortality, as well as comorbidities, and were categorized by demographics like age, gender, race/ethnicity, location, and socioeconomic status. Ethical approval was not required as this study used publicly available data and followed the Strengthening the Reporting of Observational Studies in Epidemiology (STROBE) guidelines [11].

### 2.2. Data Abstraction 

The extracted dataset incorporated variables such as year, age, gender, race, state, census region, and an urban–rural categorization. The population was segmented into three age brackets: 25–44 years, 45–64 years, and 65–85+ years of age.

The classification of geographic locations was based on states and the four census regions: Northeast, Midwest, South, and West, as outlined by the US Census Bureau [12].

Race/ethnicity was classified as non-Hispanic (NH) White, NH Black or African American, Hispanic or Latino, NH American Indian or Alaskan Native, and NH Asian or Pacific Islander. This information relies on reported data on death certificates and has been used in previous analyses of the CDC WONDER database.

The National Center for Health Statistics Urban-Rural Classification Scheme categorized the populations into urban (large metropolitan areas with ≥1 million people, medium/small metropolitan areas with 50,000–999,999 people) and rural (counties with <50,000 people) based on the 2013 U.S. census classification [13].

### 2.3. Statistical Analysis

To analyze the national trends in cirrhosis-related mortality concurrent with diabetes mellitus, age-adjusted mortality rates (AAMRs) with 95% confidence intervals (CIs) were calculated per 100,000 individuals using the 2000 US population [14] as a standard for the years 1999–2020, as used in previous studies [15]. These rates were classified according to year, sex, race/ethnicity, age, urbanization, census region, and state. 

To quantify the annual percentage change (APC) in the age-adjusted mortality rates (AAMRs) along with a 95% confidence interval, we utilized the Joinpoint Regression Program (Version 5.3.0.0, National Cancer Institute) [16]. This approach detected significant temporal changes in AAMR by applying log-linear regression models to identify periods of variation. An APC was classified as increasing or decreasing if the slope of the mortality trend was significantly different from zero, determined using two-tailed t-tests, with a *p*-value of <0.05 considered statistically significant. 

## 3. Results

Between 1999 and 2020, a total of 90,418 deaths were recorded among individuals with liver cirrhosis and diabetes mellitus. 

### 3.1. Annual Trends for Cirrhosis and Diabetes Mellitus-Related Mortality

**Figure 1:**
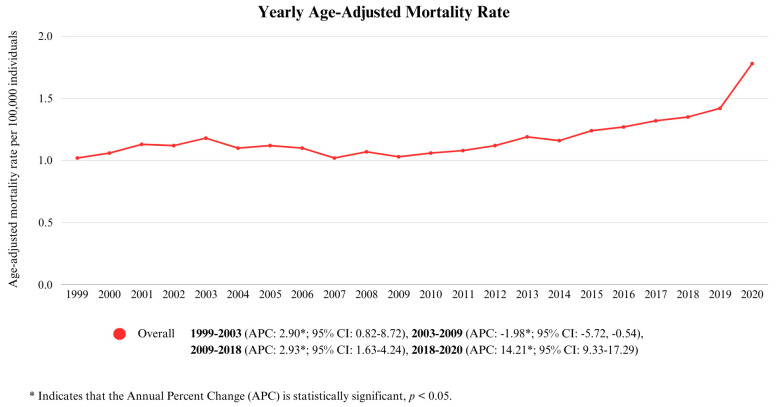
Yearly Age-Adjusted Mortality Rate.

Between 1999 and 2020, the overall age-adjusted mortality rate (AAMR) for cirrhosis and diabetes mellitus comorbid deaths among individuals of all ages was 1.21/100,000 (95% CI: 1.2–1.21). The AAMRs expressed a statistically significant increase from 1.02/100,000 (95% CI: 0.98–1.06) in 1999 to 1.78/100,000 (95% CI: 1.74–1.82) in 2020. Between 1999 and 2003, the AAMR steadily increased with an annual percentage change (APC) of 2.90 (*p* < 0.05; 95% CI: 0.82–8.72). However, from 2003 to 2009, the rate reversed course and exhibited a slight decline (APC: −1.98, *p* < 0.05; 95% CI: −5.72, −0.54). The AAMR climbed upward from 2009 to 2018 (APC: 2.93, *p* < 0.05; 95% CI: 1.63–4.24), and the trend continued as such from 2018 to 2020, with a consistent rise, culminating in a sharp increase (APC: 14.21, *p* < 0.05; 95% CI: 9.33–17.29). This is shown in Figure 1 and Supplementary Table S2.

### 3.2. Cirrhosis and Diabetes Mellitus-Related Mortality Stratified by Demographics

#### 3.2.1. Gender-Wise Analysis

Throughout the study period from 1999 to 2020, the overall AAMR for males was 1.45/100,000 (95% CI: 1.44–1.46), which was consistently higher than that of the overall AAMR of females at 0.98/100,000 (95% CI: 0.97–0.99).

In 1999, the AAMR for females was 0.86/100,000, followed by an increase until 2003, reaching 1.00/100,000 (APC: 2.88, *p* < 0.05; 95% CI: 0.73–8.37). This was followed by a statistically significant period of relative decline until 2009 (APC: −2.16, *p* < 0.05; 95% CI: −5.77, −0.79), after which a gradual upward trend was observed until 2018, with the AAMR rising from 0.87/100,000 to 1.08/100,000 (APC: 2.63, *p* < 0.05; 95% CI: 1.36–3.83). This was followed by a marked significant increase until 2020, when the AAMR peaked at 1.47/100,000 (APC: 14.35, *p* < 0.05; 95% CI: 8.00–18.18).

**Figure 2:**
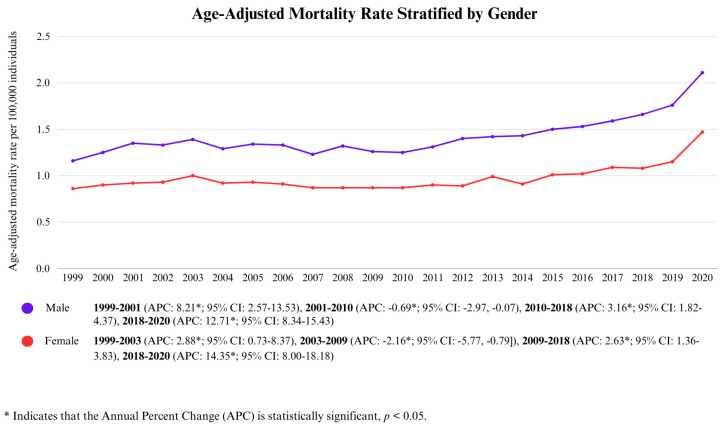
Age-Adjusted Mortality Rate Stratified by Gender.

Males exhibited only a few dissimilarities in trends with respect to female trends, beginning in 1999 with an AAMR of 1.16/100,000 (95% CI: 1.10–1.22), followed by a sharp increase until 2001 (APC: 8.21, *p* < 0.05; 95% CI: 2.57–13.53). However, from 2001 to 2010, a statistically significant decline was observed as the AAMR decreased from 1.35/100,000 (95% CI: 1.28–1.41) to 1.25/100,000 (95% CI: 1.19–1.30) (APC: −0.69, *p* < 0.05; 95% CI: −2.97, −0.07). This was followed by a moderate increase between 2010 and 2018 (APC: 3.16, *p* < 0.05; 95% CI: 1.82–4.37). Finally, from 2018 to 2020, there was a significant sharp rise, with the AAMR peaking at 2.11/100,000 (APC: 12.17, *p* < 0.05; 95% CI: 8.34–15.43). This is displayed in Figure 2 and Supplementary Table S3.

#### 3.2.2. Race-Wise Analysis

The AAMR varied significantly by race from 1999 to 2020, with notable trends emerging in different periods. The American Indian and Alaska Native population had the highest values (overall AAMR: 2.67/100,000 (95% CI: 2.54–2.81), followed by the Hispanic or Latino population (overall AAMR: 2.50/100,000; 95% CI: 2.46–2.54) and then the White population (overall AAMR: 1.23/100,000; 95% CI: 1.22–1.24). The Black or African American population and Asian or Pacific Islander population each had overall AAMRs of 0.97/100,000 (95% CI: 0.94–0.99) and 0.84/100,000 (95% CI: 0.81–0.88), respectively.

For the American Indian or Alaska Native population, the AAMRs remained relatively stable with a slight decline from 1999 to 2013 (APC: −2.59, *p* < 0.05; 95% CI: −5.93, −0.12). However, a sharp, statistically significant rise was observed from 2013 to 2020 (APC: 11.75, *p* < 0.05; 95% CI: 7.43–20.54), peaking in 2020.

The Asian or Pacific Islander population, the Black or African American population and Hispanic or Latino population exhibited a statistically significant increase in AAMRs from 2018 to 2020 (APC: 15.66, *p* < 0.05; 95% CI: 0.23–27.63), (APC: 11.23, *p* < 0.05; 95% CI: 0.73–16.59), and (APC: 15.28, *p* < 0.05; 95% CI: 3.64–20.92), respectively.

**Figure 3:**
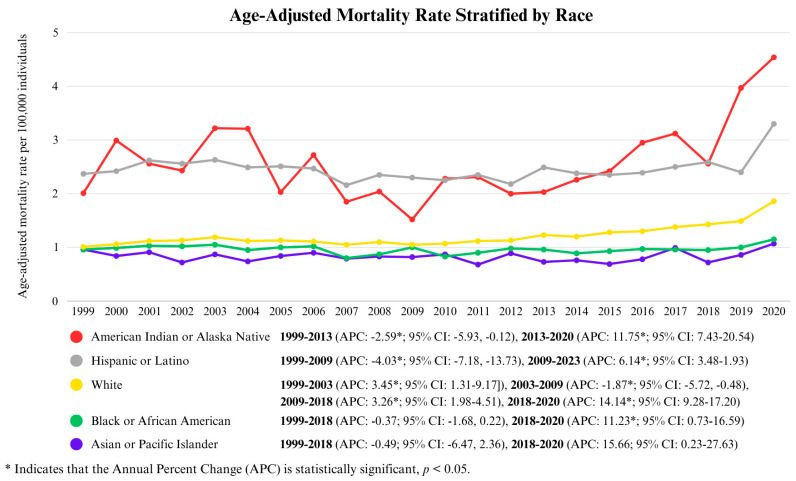
Age-Adjusted Mortality Rate Stratified by Race.

The White population exhibited different phases of change. From 1999 to 2003, AAMRs rose substantially (APC: 3.45, *p* < 0.05; 95% CI: 1.31–9.17), followed by a decline between 2003 and 2009 (APC: −1.87, *p* < 0.05; 95% CI: −5.72, −0.48). However, from 2009 to 2018, AAMRs surged once again (APC: 3.26, *p* < 0.05; 95% CI: 1.98–4.51), culminating in a sharp increase from 2018 to 2020 (APC: 14.14, *p* < 0.05; 95% CI: 9.28–17.20) (Figure 3 and Supplementary Table S4).

#### 3.2.3. Age-Wise Analysis

The overall age-adjusted mortality rates (AAMRs) for the 25–44 year, 45–64 year, and 65+ year age groups were 0.11/100,000 (95% CI: 0.10–0.11), 1.85/100,000 (95% CI: 1.83–1.87), and 6.05/100,000 (95% CI: 6.00–6.10), respectively.

To maintain consistency across geographic and condition-specific analyses, age groups were defined as 25–44, 45–64, and 65+ years, consistent with CDC WONDER standards and ensuring stable mortality estimates. Although finer age stratification might yield greater clinical insight, particularly given the early onset of cirrhosis in some cases, data for individuals under 25 were unavailable or suppressed due to low death counts. Moreover, cirrhosis and diabetes are diseases predominantly observed in older populations, which further supports the use of these broader adult age brackets for meaningful trend analysis.

For the 25–44 age group, the AAMR exhibited one distinct phase of change over the entire study period, which depicted a slight decline (APC: −0.51; 95% CI: −1.91, 0.80); this, however, was not statistically significant.

For the 45–64 age group, AAMRs showed a gradual increase over the majority of the study period. From 1999 to 2018, AAMRs exhibited a modest, statistically significant upward trend (APC: 0.93, *p* < 0.05; 95% CI: 0.12–1.44), indicating relatively stable rates with slight fluctuations. However, from 2018 to 2020, there was a sharp surge in AAMRs (APC: 12.92, *p* < 0.05; 95% CI: 3.45–18.00), marking a significant acceleration in the rising trend observed earlier.

**Figure 4:**
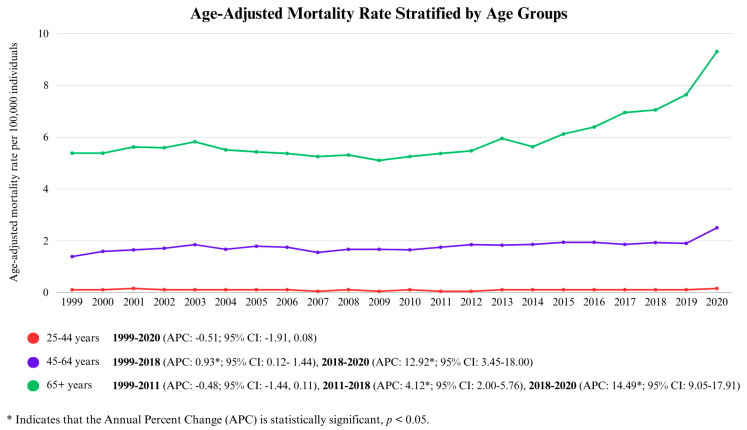
Age-Adjusted Mortality Rate Stratified by Age Groups.

For the 65+ age group, the AAMRs initially exhibited a gradual rising trend between 2011 and 2018 (APC: 4.12, *p* < 0.05; 95% CI: 2.00–5.76). The most pronounced change occurred from 2018 to 2020, when mortality rates experienced a statistically significant, sharp surge (APC: 14.49, *p* < 0.05; 95% CI: 9.05–17.91). This is shown in Figure 4 and Supplementary Table S5.

### 3.3. Cirrhosis and Diabetes Mellitus-Related Mortality Stratified by Region

#### 3.3.1. Urbanization-Wise Analysis

During the study period, both non-metropolitan and metropolitan areas exhibited a long-term upward trend in AAMRs, with non-metropolitan areas consistently showing higher values and a more pronounced increase over time.

The trajectory in metropolitan areas can be divided into four distinct phases. The initial phase (1999–2003) saw a moderate rise in AAMR (APC: 2.84, *p* < 0.05; 95% CI: 0.57–9.47), followed by a period of decline until 2009 (APC: −2.01, *p* < 0.05; 95% CI: −5.81, −0.45). From 2009 to 2018, AAMRs resumed a steady increase (APC: 2.27, *p* < 0.05; 95% CI: 0.82–3.92), culminating in a sharp surge from 2018 to 2020 (APC: 13.83, *p* < 0.05; 95% CI: 8.06–17.30), ultimately peaking at 1.66/100,000.

**Figure 5:**
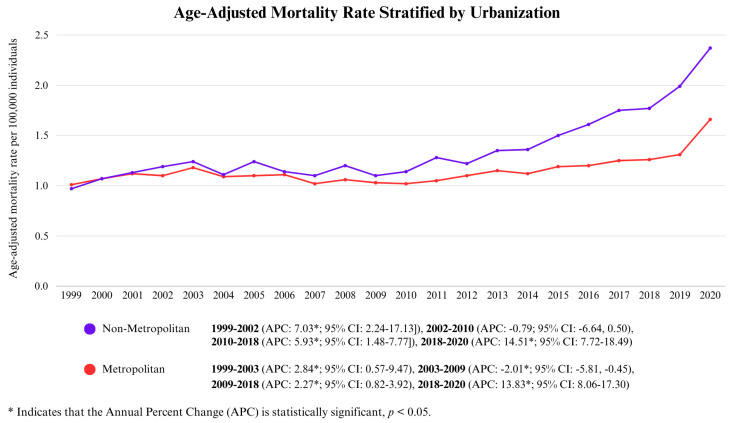
Age-Adjusted Mortality Rate Stratified by Urbanization.

In contrast, non-metropolitan areas followed a similar pattern but with a steeper and more persistent rise. The initial escalation between 1999 and 2002 was more pronounced than in metropolitan areas (APC: 7.03, *p* < 0.05; 95% CI: 2.24–17.13). This was followed by a slower but insignificant decline between 2002 and 2010 (APC: −0.79, *p* > 0.05; 95% CI: −6.64, 0.05). After 2010, AAMRs climbed at a faster pace (APC: 5.93, *p* < 0.05; 95% CI: 1.48–7.77) before experiencing an even steeper surge from 2018 to 2020 (APC: 14.51, *p* < 0.05; 95% CI: 7.72–18.49). By the end of the study period, the AAMR in non-metropolitan areas had reached 2.37. This is shown in Figure 5 and Supplementary Table S6.

#### 3.3.2. Census Region-Wise Analysis

Between 1999 and 2020, the South experienced the highest age-adjusted mortality rate (AAMR) at 1.34 (95% CI: 1.32–1.35), followed by the West at 1.33 (95% CI: 1.31–1.35), the Midwest at 1.08 (95% CI: 1.07–1.1), and finally the Northeast, at 0.92 (95% CI: 0.90–0.93).

In the South, AAMRs increased from 1999 to 2003 (APC: 5.30, *p* < 0.05; 95% CI: 2.77–11.82), with a subsequent decline from 2003 to 2007 (APC: −3.32, *p* < 0.05; 95% CI: −6.63, −0.36). This was then followed by a period of steady increase from 2007 to 2018 (APC: 3.11, *p* < 0.05; 95% CI: 2.16–4.25), after which a sharp surge was observed until 2020 (APC: 16.20, *p* < 0.05; 95% CI: 9.54–19.79). 

In the West, AAMRs showed a marked rise from 2018 to 2020 (APC: 11.26, *p* < 0.05; 95% CI: 4.17–15.89), which was the only statistically significant trend for the aforementioned census region.

The Midwest experienced an initial increase from 1999 to 2003 (APC: 3.59, *p* < 0.05; 95% CI: 0.29–14.19), followed by a decline from 2003 to 2010 (APC: −2.23, *p* < 0.05; 95% CI: −7.74, −0.64), and ultimately peaked from 2018 to 2020 (APC: 14.26, *p* < 0.05; 95% CI: 6.34–19.38).

**Figure 6:**
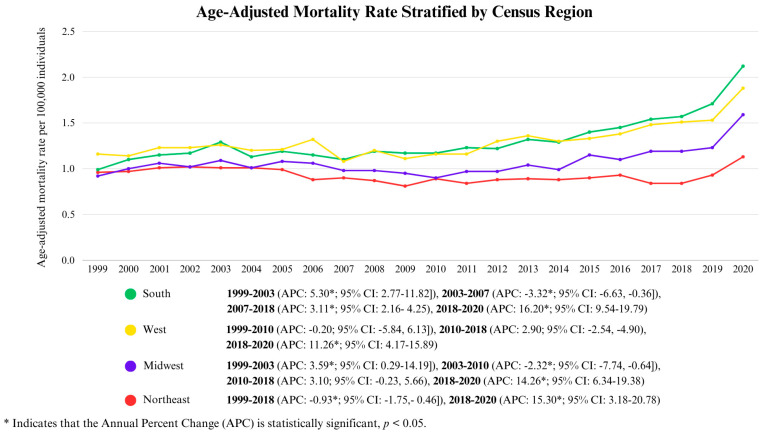
Age-Adjusted Mortality Rate Stratified by Census Region.

Lastly, in the Northeast, AAMRs declined consistently from 1999 to 2018 (APC: −0.93, *p* < 0.05; 95% CI: −1.75, −0.46) but then experienced a quick rise from 2018 to 2020 (APC: 15.30, *p* < 0.05; 95% CI: 3.18–20.78). This is displayed in Figure 6 and Supplementary Table S7.

#### 3.3.3. State Wise Analysis

Age-adjusted mortality rates (AAMRs) for the 50 states and the District of Columbia revealed considerable geographic variation.

**Figure 7:**
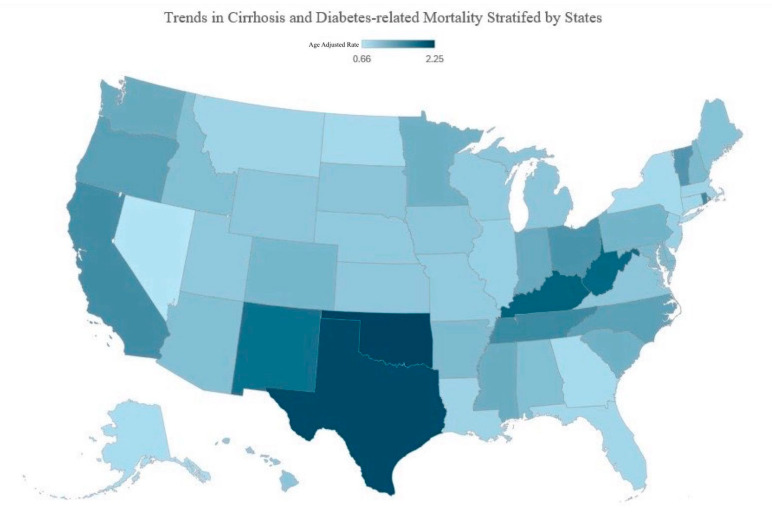
Age-Adjusted Mortality Rate Stratified by State.

Oklahoma (2.25/100,000), Texas (2.20/100,000), and Kentucky (1.97/100,000) ranked in the top 90th percentile in terms of AAMRs. Moderate AAMRs were seen in states such as Tennessee (1.57/100,000) and Rhode Island (1.59/100,000), where mortality rates remained elevated but were not as extreme as those in the highest-ranking states. In contrast, lower AAMRs were found in Nevada (0.66/100,000) and Georgia (0.77/100,000), ranking in the bottom 10th percentile (Figure 7 and Supplementary Table S8).

## 4. Discussion

We report several significant findings in the 21-year analysis of the CDC-WONDER database primarily on mortality due to concurrent DM and liver cirrhosis occurring between 1999 and 2020. This comprehensive analysis has been stratified by year, age, gender, race, state, census region, and an urban–rural categorization, interpreting the most salient findings and situating them in the context of biological, clinical, and public health frameworks.

**Figure 8:**
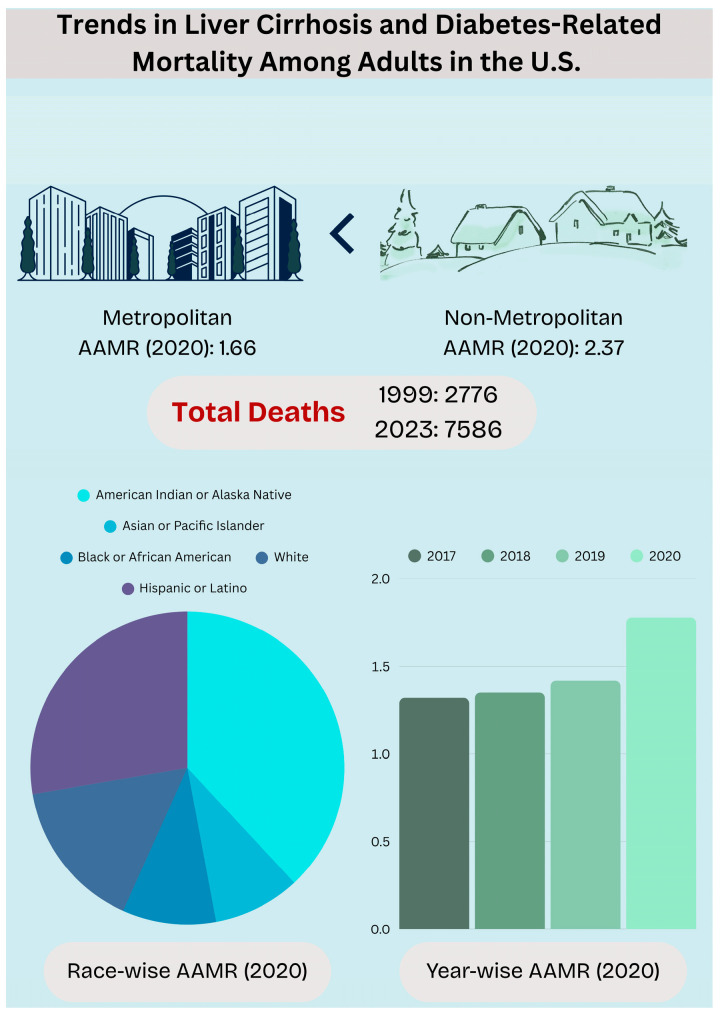
Trends in Liver Cirrhosis and Diabetes-Related Mortality Among Adults in the U.S.

Figure 8 summarizes the key supplementary data.

Current literature strongly links Cirrhosis to glucose metabolism disorders. About 30% of patients have overt diabetes, and 30–50% exhibit impaired tolerance [17,18,19,20,21]. Where insulin resistance is from reduced hepatic clearance [22] and portosystemic shunting [17], combined with inflammation [23], drives both type 2 and hepatogenous diabetes while accelerating fibrosis. Moreover, metabolic syndrome combined with cytokine-driven stress amplifies cirrhosis [24,25,26]. These issues foster a persistent cycle of liver damage and disrupted glucose regulation.

In cirrhosis, diabetes may be pre-existing type 2 DM or develop later as hepatogenous diabetes, which arises due to portal hypertension and liver dysfunction. Type 2 diabetes, a key part of metabolic syndrome, promotes non-alcoholic fatty liver disease (NAFLD) [27] and independently accelerates fibrosis through multiple mechanisms. Cirrhosis disrupts glucose regulation [8] by causing insulin resistance in muscle and fat, leading to hyperinsulinemia [28]. Liver dysfunction reduces insulin clearance [22] and portosystemic shunting lowers hepatic insulin breakdown, raising systemic insulin levels [17]. This worsens insulin signaling, making impaired hepatic glucose uptake the main cause of hyperglycemia in cirrhotic patients [29].

The majority of diabetic cirrhotic individuals have type 2 DM, which is caused by beta-cell failure and insulin resistance and has common risk factors that connect it with NAFLD and cirrhosis. Type 1 DM is uncommon and is caused by autoimmune beta-cell destruction [30].

Cirrhosis progresses through chronic inflammation [23] and mitochondrial stress, boosted by cytokines from fat and insulin resistance. Stellate cells induce fibrosis by excessive production of collagen. Hyperglycemia and oxidative stress enhance advanced glycation end products (AGE) [31] accumulation, perpetuating fibrosis, and liver damage.

While the increase in AAMR from 1.02 to 1.78 per 100,000 may appear modest, it reflects a meaningful clinical burden. In 2019, diabetes and chronic liver disease accounted for approximately 2.6 million and 1.8 million disability-adjusted life years (DALYs) in the U.S., respectively, while cardiovascular diseases exceeded 17 million DALYs. These figures emphasize that even small rises in mortality can have significant public health implications, particularly for aging and high-risk populations [32,33,34].

The mortality trends indicate a significant modest increase from 1999 to 2003, followed by a slight significant decline until 2009. Although the literature addressing the initial upward trend is limited, potential contributing factors may include the increasing prevalence of chronic conditions such as obesity and hypercholesterolemia, constraints within healthcare delivery during the early 2000s, and possible modifications in disease classification that could affect reporting accuracy. This trend may also have been influenced by factors such as heightened alcohol consumption and the widespread occurrence of hepatitis infections, while the decline in the following can be said to be due to the improvements in medical treatments, early detection, and public health initiatives likely leading to a decrease in mortality rates from chronic liver diseases and cirrhosis during this period [27]. The period from 2018 to 2020 saw the most significant rise in mortality, an effect that is plausibly related to the disruptive impact of the COVID-19 pandemic. According to a comprehensive analysis, an estimated 18.2 million excess deaths occurred globally from 1 January 2020, to 31 December 2021. This excess mortality reflects not only direct COVID-19 deaths but also indirect effects such as healthcare disruptions and socioeconomic instability [35]. The pandemic not only overwhelmed healthcare systems, resulting in delayed care and diagnoses, but also had broader repercussions, including increased mental health challenges, economic stress, and suboptimal allocation of healthcare resources [36]. Given these global findings, it is plausible that the rise in mortality observed in our data is at least partially attributable to the pandemic.

Out of the three age groups studied, the mortality rate showed a statistically insignificant decline for ages 25–44 years during the years 1999–2020. The trend can be due to the increase in public health efforts and better approaches to treat diabetes that help manage and prevent complications [37]. Two significant trends were observed for the age group of 45–64 years, including a significant marked rise from 2018 to 2020. The rise points towards the growing burden of cirrhosis from non-alcoholic fatty liver disease (NAFLD) and diabetic complications [38]. It might also be linked to worsening metabolic syndrome and the increasing prevalence of advanced fibrosis in diabetics [39,40]. However, diabetes remains a significant health concern among older adults. Moreover, the coexistence of diabetes and liver disease can exacerbate health outcomes. Diabetes is a known risk factor for the development and progression of NAFLD, which can advance to cirrhosis and contribute to the mortality trend. Moreover, there is an increased prevalence of comorbidities in this age group, such as cardiovascular diseases (CVDs), and CVD is the primary cause of mortality in individuals with both NAFLD and type 2 DM [41,42]. The combination of these conditions in older adults can lead to increased morbidity and mortality, as can be seen in the mortality trends of 2011–2018, with a significant moderate increase followed by a significant rapid rise from 2018 to 2020. Furthermore, several studies report that estrogen exerts a protective effect against both diabetes and cirrhosis, hence, one can argue that the overall highest trend being observed in older age groups also owes to a risk of these diseases in post-menopausal females [43,44]. The sharp increase across all age groups from 2018–2020 might reflect underlying diabetes-related immunosuppression and cirrhosis-related vulnerability to severe infections, including COVID-19 [45].

The AAMR values varied across states, underscoring the influence of regional health determinants. The South and Midwest exhibit the highest mortality rates, particularly Oklahoma and Texas, which exhibited the greatest mortality rates (≥2.0), reflecting metabolic risk factors and healthcare access disparities, such as access and distribution [46,47,48]. These states also have some of the highest obesity and diabetes rates, which are key risk factors for cirrhosis progression [47]. In contrast, Northeastern states exhibit lower rates, likely due to superior healthcare infrastructure and lower obesity prevalence, which in turn reduces metabolic disease burden and cirrhosis risk [46,47]. The West is variable, with California and Oregon showing elevated rates, possibly due to growing Hispanic populations at risk for NAFLD. Furthermore, Nevada, Georgia, and Alaska maintain lower mortality rates (≤1.0), potentially reflecting better healthcare access. Moreover, states such as New York, Massachusetts, and Connecticut benefit from robust healthcare systems and effective liver disease management, a fact underscored by their top rankings in the Commonwealth Fund’s 2020 Scorecard [49].

We also identified notable disparities based on sex and race. Our 21-year analysis revealed a significant amplification in AAMR for both genders. However, men accounted for the larger proportion of cirrhosis-related deaths among patients with DM.

This disproportionate trend can be justified by several factors. Men generally consume more alcohol than women, increasing the risk of alcohol-related liver diseases such as cirrhosis [50]. Moreover, estrogen in women has been found to have a protective role against various liver diseases in women [43,44]. Men are typically less proactive in seeking regular medical care, often resulting in delayed diagnoses and treatment of conditions such as diabetes and liver disease, ultimately increasing their risk of mortality [51].

NH American Indian or Alaska Natives (AI/AN) had the highest mortality rates, followed by Hispanics or Latinos, while Asian populations exhibited the lowest. This disparity in cirrhosis-DM-related mortality amongst different races can be attributed to several factors.

AI/AN populations have significantly higher rates of sugar-sweetened beverage consumption, obesity, diabetes, hypertension, and physical inactivity compared to white populations. AI/AN individuals have reported having less access to a healthcare provider (63.1%) than other races (72.8%). These disparities contribute to the higher rates of cirrhosis and diabetes-related mortality in AI/AN populations. The combination of dietary habits, obesity, chronic disease burden, and limited healthcare access exacerbates health outcomes, leading to greater mortality from preventable conditions [52]. Social factors contributing to escalated mortality in NH AI/AN groups include language barriers, poverty, decreased access to health insurance, and racial discrimination, in addition to having the lowest educational attainment rates compared to all other ethnic groups in the United States [53].

The relatively low prevalence of alcoholic liver disease among Asian populations has likely played a role in the observed differences in cirrhosis-related mortality within these groups [54,55,56]. Moreover, genome-wide association studies have identified the patatin-like phospholipase domain-containing-3 (PNPLA3) gene as a key factor in hepatic fat accumulation and hepatocyte damage [57]. This gene is strongly linked to alcohol-associated liver disease (ALD). It is more prevalent among Hispanic populations, contributing to higher rates of non-alcoholic fatty liver disease (NAFLD) and potentially explaining the high prevalence of ALD in this group [58,59], leading to glucose intolerance. The disparity in cirrhosis–diabetes-related mortality among different racial and ethnic groups can be attributed to a combination of socioeconomic and healthcare access factors. For example, NH American Indian or Alaska Native (AI/AN) populations have significantly lower access to healthcare providers (63.1%) compared to other groups (72.8%), compounded by higher rates of poverty, food insecurity, and geographic isolation. Hispanic and Black populations also face disproportionately high rates of being uninsured or underinsured, leading to delays in diagnosis and treatment. According to recent national estimates, Hispanic adults have the highest uninsured rates (about 27%), followed by Black adults (15%), compared to White adults (9%). These barriers, along with differences in health literacy, transportation access, and cultural mistrust of the medical system, exacerbate chronic disease outcomes and contribute to elevated mortality rates in these populations [60].

We observed that in recent years, both metropolitan and non-metropolitan areas have experienced rising AAMRs for cirrhosis and DM-related deaths. However, non-metropolitan regions have seen a markedly steeper increase after 2010, deepening the mortality gap between urban and rural communities. 

Key factors contributing to rural health disparities include geographic isolation, lower income levels, limited access to specialists, and fewer job opportunities. Residents of most rural areas do not have employer-provided health insurance. Moreover, cultural attitudes towards healthcare and disease prevention may differ in non-metropolitan areas, influencing health behaviors and engagement with medical services. There is also a delayed detection of diseases in rural areas due to a lack of screening and early detection of diseases, resulting in worsened health outcomes [61]. Furthermore, a recent study found that patients with DM face an increased risk of liver decompensation associated with rural living, lower socioeconomic status, and neighborhood poverty. To explore these relationships, the researchers used a multivariable analysis with Cox proportional hazards and Fine–Gray competing risk models. Neighborhood-level indicators, such as poverty, affluence, and disadvantage were used to assess how well-off or deprived an area is, while rurality was measured using a scoring system that reflects how rural or urban a location is. The statistical models helped evaluate how these factors influence the risk of liver decompensation and other health outcomes over time, taking into account both timing and competing risks [62].

### 4.1. Clinical Implications

The above discussion highlights the necessity of targeted interventions to reduce mortality trends associated with coexistent diabetes and cirrhosis. Advancements in diagnostic approaches, such as non-invasive biomarker quantification [63], elastography [64], and MRI-based liver stiffness assessment, have significantly improved the early detection of liver fibrosis, preventing further deterioration in glucose homeostasis and reducing the risk of hepatogenous diabetes. The diagnosis of diabetes in cirrhotic patients remains challenging due to altered glucose metabolism. While fasting plasma glucose, A1C, and the oral glucose tolerance test (OGTT) are useful in compensated cirrhosis [65], A1C may be unreliable in decompensated cases due to anemia, portal hypertension, and chronic kidney disease. OGTT is the preferred diagnostic test, while continuous glucose monitoring (CGM) offers real-time insights into glucose fluctuations [66].

Early interventions are crucial given future projections of increasing disease mortality, as described previously. The Child–Pugh scoring system [67] helps guide treatment strategies based on hepatic function, and therapeutic approaches must consider renal function, prognosis, and complications like ascites. Lifestyle modifications remain the first-line recommendation. Metformin is the preferred treatment per the American Diabetes Association [68]. Insulin remains the mainstay for advanced cirrhosis [69]. Liver transplantation has greatly improved survival in decompensated cirrhosis [70] and restored glucose tolerance and insulin sensitivity [71]. However, many cirrhotic patients die on the transplant list due to a shortage of donors and the inability to accurately predict life expectancy [72].

### 4.2. Limitations

Several limitations should be considered when evaluating this study. Firstly, the analysis depends on the accuracy and completeness of data within the CDC WONDER database. Since multiple factors may contribute to a person’s death, distinguishing the primary cause from contributing factors can be challenging. Additionally, the database only provides information on patient age, gender, ethnicity/race, geographic location, and level of urbanization, meaning that there are no data on co-morbidities, insurance coverage, or medication history. Furthermore, as death certificates rather than full patient records are used, cases of DM may have been diagnosed but not documented on the death certificate, potentially leading to underreporting of COPD-related mortality in diabetic patients. Migration factors may influence data, as patients travelling across regions with concomitant diseases and passing away in another region can skew the data that have been entered into the national database. The National Center for Health Statistics (NCHS) rural–urban classification oversimplifies the complex socioeconomic diversity within these areas. For instance, affluent suburbs and impoverished inner-city neighborhoods—both labeled “urban”—likely differ in health outcomes and healthcare use. Future research should use more precise socioeconomic indicators to capture these within-category differences. An additional limitation of our study is the inability to perform multivariable-adjusted analyses, as the CDC WONDER database does not provide individual-level data. This restricts our capacity to control for key confounders, such as socioeconomic status, comorbidities, insurance coverage, or healthcare access. As a result, observed disparities, particularly by race, region, or urbanization, cannot be fully interpreted as independent effects. Future research using datasets with richer sociodemographic and clinical variables is needed to clarify these associations through adjusted modeling approaches. The accuracy of cause-of-death data may be affected by ICD-10 coding changes and misclassification over time. Additionally, CDC WONDER lacks data on key confounders, such as obesity, alcohol use, and viral hepatitis, which may influence mortality trends and limit causal interpretation.

## 5. Conclusions

Approximately 178,000 people die due to excessive drinking every year in the United States [73]. Heavy alcohol abuse diminishes the function of the liver and the pancreas, which in turn affects glucose control in the body. Diabetes worsens the prognosis of cirrhosis and accelerates liver fibrosis, increasing mortality rates. Effective treatment should focus on breaking this cycle to improve outcomes. Addressing both cirrhosis and diabetes calls for a multifaceted approach. By using non-invasive testing, individualized treatment plans, reliable biomarkers, and advanced imaging, the early identification and regular monitoring of both conditions become feasible. In addition, more comprehensive clinical trials are necessary to verify the effectiveness of new treatments, while greater emphasis on structured weight loss initiatives, anti-obesity drugs, and bariatric surgery could improve long-term outcomes.

In summary, the intersection of cirrhosis and diabetes necessitates a refined understanding of diagnostic limitations and therapeutic options. Ongoing research alongside a collaborative care model is pivotal in enhancing patient outcomes in this complex clinical scenario.

## Data Availability

The data presented in this study are openly available in [CDC Wonder] at https://wonder.cdc.gov/, accessed on 16 January 2025.

## Supplementary Materials

The following supporting information can be downloaded at: https://www.mdpi.com/article/10.3390/life15060852/s1, Table S1: The 10th International Classification of Diseases (ICD-10) pertaining to both Cirrhosis and Diabetes included in our study. Table S2: Overall Cirrhosis-related AAMR in patients with DM per 100,000 in the United States from 1999 to 2020. Table S3: Sex-stratified Cirrhosis-related AAMR in patients with DM per 100,000 in the United States from 1999 to 2020. Table S4: Cirrhosis-related Age-Adjusted Mortality Rates in patients with DM per 100,000 in the United States from 1999 to 2020 stratified by race. Table S5: Cirrhosis-related Crude Rate in patients with DM per 100,000 in the United States from 1999 to 2020 stratified by age groups. Table S6: Cirrhosis related mortality AAMR in patients with DM per 100,000 stratified by Urban-Rural classification in the United States from 1999 to 2020. Table S7: Cirrhosis related mortality AAMR in patients with DM per 100,000 stratified by census region in the United States from 1999 to 2020. Table S8: Cirrhosis related mortality AAMR in patients with DM per 100,000 stratified by state in the United States from 1999 to 2020. Table S9: Summary APCs of Cirrhosis-related AAMR in Patients with Diabetes Mellitus per 100,000 in the United States from 1999 to 2020.
