# Supplementary material for: Trends in Liver Cirrhosis and Diabetes-Related Mortality Among Adults in the United States: A CDC WONDER Database Analysis (1999–2020)
Source: Life (Basel). 2025 May 25;15(6):852. doi: 10.3390/life15060852 (PMC12194481; doi:10.3390/life15060852)
Supplement: Supplementary file 1 [file life-15-00852-s001.zip › life-3633433-supplementary.pdf]

[*Supplementary Material*]

Trends in Vascular Dementia-Related Mortality

In the United States from 2005 to 2020

Diabetes and Cirrhosis	ICD-10 Code
Type 1 diabetes mellitus	E10
Type 2 diabetes mellitus	E11
Malnutrition-related diabetes mellitus	E12
Other specified diabetes mellitus	E13
Unspecified diabetes mellitus	E14
Pre-existing diabetes mellitus, insulin-dependent	O24.0
Pre-existing diabetes mellitus, non-insulin-dependent	O24.1
Pre-existing malnutrition-related diabetes mellitus	O24.2
Pre-existing diabetes mellitus, unspecified	O24.3
Fibrosis and cirrhosis of liver	K74

Supplementary Table S1. Includes all codes from the 10th International Classification of Diseases (ICD-10) pertaining to both Cirrhosis and Diabetes included in our study

Year	Overall<br>Age Adjusted Rate Mortality Rate (95% CI)
1999	1.02 (098-1.06)
2000	1.06 (1.03-1.1)
2001	1.13 (1.09-1.16)
2002	1.12 (1.08-1.16)
2003	1.18 (1.14-1.22)
2004	1.1 (1.06-1.14)
2005	1.12 (1.08-1.15)
2006	1.1 (1.06-1.14)
2007	1.02 (0.99-1.06)
2008	1.07 (1.04-1.11)
2009	1.03 (1.00-1.07)
2010	1.06 (1.03-1.1)
2011	1.08 (1.05-1.12)
2012	1.12 (1.08-1.15)
2013	1.19 (1.15-1.23)
2014	1.16 (1.12-1.19)
2015	1.24 (1.2-1.27)
2016	1.27 (1.24-1.31)
2017	1.32 (1.29-1.36)
2018	1.35 (1.32-1.39)
2019	1.42 (1.38-1.46)
2020	1.78 (1.74-1.82)

Supplementary Table S2. Overall Cirrhosis-related AAMR in patients with DM per 100,000 in the United States from 1999 to 2020

Gender	Year	Age Adjusted Mortality Rate (95% CI)
**Female**	1999	0.86 (0.82-0.91)
**Female**	2000	0.9 (0.85-0.95)
**Female**	2001	0.92(0.87-0.97)
**Female**	2002	0.93 (0.88-0.98)
**Female**	2003	1 (0.95-1.05)
**Female**	2004	0.92(0.88-0.97)
**Female**	2005	0.93 (0.88-0.97)
**Female**	2006	0.91 (0.86-0.95)
**Female**	2007	0.87 (0.82-0.91)
**Female**	2008	0.87 (0.83-0.92)
**Female**	2009	0.87 (0.82-0.91)
**Female**	2010	0.87 (0.82-0.91)
**Female**	2011	0.9 (0.85-0.94)
**Female**	2012	0.89 (0.85-0.94)
**Female**	2013	0.99 (0.94-1.03)
**Female**	2014	0.91 (0.87-0.95)
**Female**	2015	1.01 (0.97-1.06)
**Female**	2016	1.02 (0.98-1.07)
**Female**	2017	1.09 (1.04-1.13)
**Female**	2018	1.08 (1.04-1.13)
**Female**	2019	1.15 (1.1-1.19)
**Female**	2020	1.47 (1.42-1.52)
**Male**	1999	1.16 (1.1-1.22)
**Male**	2000	1.25 (1.19-1.32)
**Male**	2001	1.35 (1.28-1.41)
**Male**	2002	1.33 (1.26-1.39)
**Male**	2003	1.39 (1.32-1.45)
**Male**	2004	1.29 (1.23-1.35)
**Male**	2005	1.34 (1.28-1.4)
**Male**	2006	1.33 (1.27-1.39)
**Male**	2007	1.23 (1.17-1.29)
**Male**	2008	1.32 (1.26-1.38)
**Male**	2009	1.26 (1.2-1.32)

**Male**	2010	1.25 (1.19-1.3)
**Male**	2011	1.31 (1.2-1.36)
**Male**	2012	1.4 (1.34-1.46)
**Male**	2013	1.42 (1.36-1.48)
**Male**	2014	1.43 (1.37-1.48)
**Male**	2015	1.5 (1.44-1.56)
**Male**	2016	1.53 (1.48-1.59)
**Male**	2017	1.59 (1.54-1.65)
**Male**	2018	1.66 (1.6-1.71)
**Male**	2019	1.76 (1.7-1.82)
**Male**	2020	2.11 (2.05-2.18)

Supplementary Table S3. Sex-stratified Cirrhosis-related AAMR in patients with DM per 100,000 in the United States from 1999 to 2020

Age Adjusted Mortality Rate (95%CI)

Year	NH American Indian	NH Asian	NH Black	NH White	Hispanic
**1999**	2.01 (1.63-2.39)	0.96 (0.84-1.08)	0.96 (0.90-1.02)	1.01 (0.97-1.05)	2.37 (2.11-2.63)
**2000**	2.99 (2.54-3.44)	0.84 (0.73-0.95)	0.99 (0.93-1.05)	1.06 (1.02-1.10)	2.42 (2.16-2.68)
**2001**	2.56 (2.15-2.97)	0.91 (0.80-1.02)	1.03 (0.97-1.09)	1.12 (1.08-1.16)	2.62 (2.36-2.88)
**2002**	2.43 (2.06-2.80)	0.72 (0.62-0.82)	1.02 (0.96-1.08)	1.13 (1.09-1.17)	2.56 (2.30-2.82)
**2003**	3.22 (2.79-3.65)	0.87 (0.77-0.97)	1.05 (0.99-1.11)	1.19 (1.15-1.23)	2.63 (2.37-2.89)
**2004**	3.21 (2.79-3.63)	0.74 (0.65-0.83)	0.95 (0.89-1.01)	1.12 (1.08-1.16)	2.49 (2.23-2.75)
**2005**	2.03 (1.72-2.34)	0.84 (0.75-0.93)	1.00 (0.94-1.06)	1.13 (1.09-1.17)	2.51 (2.25-2.77)
**2006**	2.72 (2.35-3.09)	0.90 (0.81-0.99)	1.02 (0.96-1.08)	1.11 (1.07-1.15)	2.47 (2.21-2.73)
**2007**	1.85 (1.55-2.15)	0.79 (0.70-0.88)	0.80 (0.74-0.86)	1.05 (1.01-1.09)	2.16 (1.92-2.40)
**2008**	2.04 (1.74-2.34)	0.83 (0.74-0.92)	0.87 (0.81-0.93)	1.10 (1.06-1.14)	2.35 (2.11-2.59)
**2009**	1.52 (1.26-1.78)	0.82 (0.73-0.91)	1.00 (0.94-1.06)	1.05 (1.01-1.09)	2.30 (2.06-2.54)
**2010**	2.28 (1.96-2.60)	0.87 (0.79-0.95)	0.83 (0.77-0.89)	1.07 (1.03-1.11)	2.25 (2.01-2.49)
**2011**	2.31 (1.99-2.63)	0.68 (0.61-0.75)	0.90 (0.84-0.96)	1.12 (1.08-1.16)	2.35 (2.11-2.59)
**2012**	2.00 (1.72-2.28)	0.89 (0.81-0.97)	0.98 (0.92-1.04)	1.13 (1.09-1.17)	2.18 (1.94-2.42)
**2013**	2.03 (1.75-2.31)	0.73 (0.66-0.80)	0.96 (0.90-1.02)	1.23 (1.19-1.27)	2.49 (2.25-2.73)
**2014**	2.26 (1.99-2.53)	0.76 (0.69-0.83)	0.89 (0.83-0.95)	1.20 (1.16-1.24)	2.38 (2.14-2.62)
**2015**	2.42 (2.16-2.68)	0.69 (0.63-0.75)	0.93 (0.87-0.99)	1.28 (1.24-1.32)	2.35 (2.11-2.59)
**2016**	2.95 (2.65-3.25)	0.78 (0.71-0.85)	0.97 (0.91-1.03)	1.30 (1.26-1.34)	2.39 (2.15-2.63)
**2017**	3.12 (2.82-3.42)	0.99 (0.92-1.06)	0.96 (0.90-1.02)	1.38 (1.34-1.42)	2.50 (2.26-2.74)
**2018**	2.56 (2.30-2.82)	0.72 (0.66-0.78)	0.95 (0.89-1.01)	1.43 (1.39-1.47)	2.59 (2.35-2.83)
**2019**	3.97 (3.61-4.33)	0.86 (0.79-0.93)	1.00 (0.94-1.06)	1.49 (1.45-1.53)	2.40 (2.16-2.64)
**2020**	4.54 (4.18-4.90)	1.07 (0.99-1.15)	1.15 (1.09-1.21)	1.86 (1.82-1.90)	3.30 (3.02-3.58)

Supplementary Table S4. Cirrhosis-related Age Adjusted Mortality Rates in patients with DM per 100,000 in the United States from 1999 to 2020 stratified by race.

Year	Age Groups<br>Age Adjusted Mortality Rate (95% CI)		
	25-44	45-64	65+
1999	0.11 (0.09-0.14)	1.39 (1.29-1.48)	5.38 (5.14-5.63)
2000	0.11 (0.09-0.14)	1.59 (1.49-1.69)	5.38 (5.13-5.62)
2001	0.16 (0.13-0.19)	1.65 (1.55-1.75)	5.62 (5.37-5.87)
2002	0.11 (0.09-0.13)	1.71 (1.61-1.81)	5.59 (5.34-5.83)
2003	0.11 (0.09-0.13)	1.85 (1.75-1.95)	5.82 (5.58-6.07)
2004	0.11 (0.09-0.13)	1.67 (1.58-1.77)	5.51 (5.27-5.75)
2005	0.11 (0.09-0.13)	1.79 (1.69-1.89)	5.43 (5.19-5.67)
2006	0.11 (0.09-0.13)	1.75 (1.66-1.84)	5.37 (5.13-5.61)
2007	0.05 (0.04-0.07)	1.55 (1.46-1.64)	5.25 (5.01-5.48)
2008	0.11 (0.09-0.14)	1.67 (1.58-1.76)	5.31 (5.08-5.54)
2009	0.05 (0.04-0.07)	1.67 (1.58-1.76)	5.1 (4.88-5.32)
2010	0.11 (0.08-0.14)	1.65 (1.56-1.73)	5.25 (5.02-5.48)
2011	0.05 (0.04-0.07)	1.75 (1.66-1.83)	5.37 (5.15-5.6)
2012	0.05 (0.04-0.07)	1.85 (1.76-1.94)	5.47 (5.24-5.69)
2013	0.11 (0.08-0.14)	1.83 (1.74-1.91)	5.95 (5.72-6.18)
2014	0.11 (0.08-0.14)	1.86 (1.77-1.95)	5.63 (5.41-5.85)
2015	0.11 (0.09-0.14)	1.94 (1.85-2.03)	6.12 (5.9-6.35)
2016	0.11 (0.09-0.14)	1.94 (1.85-2.03)	6.39 (6.16-6.62)
2017	0.11 (0.09-0.14)	1.86 (1.78-1.95)	6.95 (6.71-7.18)
2018	0.11 (0.09-0.14)	1.93 (1.83-2.02)	7.05 (6.82-7.28)
2019	0.11 (0.08-0.14)	1.9 (1.81-1.99)	7.64 (7.4-7.87)
2020	0.16 (0.13-0.19)	2.5 (2.4-2.6)	9.3 (9.04-9.55)

Supplementary Table S5. Cirrhosis-related Crude Rate in patients with DM per 100,000 in the United States from 1999 to 2020 stratified by age groups. .

	Age Adjusted Mortality Rate (95% CI)	
**Year**	**Metropolitan**	**Non-Metropolitan**
**1999**	1.01 (0.96-1.05)	0.97 (0.88-1.05)
**2000**	1.07 (1.03-1.11)	1.07 (0.98-1.17)
**2001**	1.12 (1.08-1.16)	1.13 (1.03-1.22)
**2002**	1.1 (1.06-1.14)	1.19 (1.1-1.29)
**2003**	1.18 (1.13-1.22)	1.24 (1.15-1.34)
**2004**	1.09 (1.05-1.13)	1.11 (1.02-1.2)
**2005**	1.1 (1.05-1.14)	1.24 (1.15-1.34)
**2006**	1.11 (1.07-1.15)	1.14 (1.05-1.23)
**2007**	1.02 (0.98-1.06)	1.1 (1.01-1.19)
**2008**	1.06 (1.02-1.1)	1.2 (1.11-1.29)
**2009**	1.03 (0.99-1.07)	1.1 (1.02-1.19)
**2010**	1.02 (0.99-1.06)	1.14 (1.05-1.23)
**2011**	1.05 (1.01-1.09)	1.28 (1.18-1.37)
**2012**	1.1 (1.06-1.13)	1.22 (1.13-1.31)
**2013**	1.15 (1.12-1.19)	1.35 (1.25-1.44)
**2014**	1.12 (1.09-1.16)	1.36 (1.27-1.46)
**2015**	1.19 (1.16-1.23)	1.5 (1.4-1.6)
**2016**	1.2 (1.16-1.24)	1.61 (1.51-1.71)
**2017**	1.25 (1.21-1.28)	1.75 (1.65-1.86)
**2018**	1.26 (1.23-1.3)	1.77 (1.67-1.87)
**2019**	1.31 (1.27-1.35)	1.99 (1.88-2.1)
**2020**	1.66 (1.62-1.7)	2.37 (2.25-2.49)

Supplementary Table S6. Cirrhosis related mortality AAMR in patients with DM per 100,000 stratified by Urban-Rural classification in the United States from 1999 to 2020.

Census Region	Year	Age Adjusted Mortality Rate (95% CI)
**Census Region 1: South**	1999	0.99 (0.93-1.05)
**Census Region 1: South**	2000	1.1 (1.04-1.17)
**Census Region 1: South**	2001	1.15 (1.08-1.22)
**Census Region 1: South**	2002	1.17 (1.1-1.23)
**Census Region 1: South**	2003	1.29 (1.22-1.36)
**Census Region 1: South**	2004	1.13 (1.07-1.2)
**Census Region 1: South**	2005	1.19 (1.12-1.25)
**Census Region 1: South**	2006	1.15 (1.09-1.21)
**Census Region 1: South**	2007	1.1 (1.04-1.16)
**Census Region 1: South**	2008	1.19 (1.12-1.25)
**Census Region 1: South**	2009	1.17 (1.11-1.23)
**Census Region 1: South**	2010	1.17 (1.11-1.23)
**Census Region 1: South**	2011	1.23 (1.17-1.29)
**Census Region 1: South**	2012	1.22 (1.16-1.28)
**Census Region 1: South**	2013	1.32 (1.26-1.38)
**Census Region 1: South**	2014	1.29 (1.23-1.35)
**Census Region 1: South**	2015	1.4 (1.34-1.46)
**Census Region 1: South**	2016	1.45 (1.38-1.51)
**Census Region 1: South**	2017	1.54 (1.47-1.6)
**Census Region 1: South**	2018	1.57 (1.51-1.63)
**Census Region 1: South**	2019	1.71 (1.65-1.78)
**Census Region 1: South**	2020	2.12 (2.05-2.2)
**Census Region 2: West**	1999	1.16 (1.07-1.25)
**Census Region 2: West**	2000	1.14 (1.05-1.23)
**Census Region 2: West**	2001	1.23 (1.14-1.32)
**Census Region 2: West**	2002	1.23 (1.14-1.32)
**Census Region 2: West**	2003	1.26 (1.17-1.35)
**Census Region 2: West**	2004	1.2 (1.11-1.28)
**Census Region 2: West**	2005	1.21 (1.13-1.3)
**Census Region 2: West**	2006	1.32 (1.23-1.41)
**Census Region 2: West**	2007	1.08 (1-1.16)
**Census Region 2: West**	2008	1.2 (1.11-1.28)
**Census Region 2: West**	2009	1.11 (1.04-1.19)
**Census Region 2: West**	2010	1.16 (1.08-1.24)
**Census Region 2: West**	2011	1.16 (1.08-1.24)
**Census Region 2: West**	2012	1.3 (1.22-1.38)
**Census Region 2: West**	2013	1.36 (1.28-1.45)
**Census Region 2: West**	2014	1.3 (1.23-1.38)
**Census Region 2: West**	2015	1.33 (1.25-1.41)
**Census Region 2: West**	2016	1.38 (1.3-1.46)
**Census Region 2: West**	2017	1.48 (1.4-1.56)
**Census Region 2: West**	2018	1.51 (1.43-1.59)
**Census Region 2: West**	2019	1.53 (1.45-1.61)
**Census Region 2: West**	2020	1.88 (1.8-1.97)
**Census Region 3: Midwest**	1999	0.92 (0.85-1)
**Census Region 3: Midwest**	2000	1 (0.92-1.08)

**Census Region 3: Midwest**	2001	1.06 (0.98-1.14)
**Census Region 3: Midwest**	2002	1.02 (0.94-1.1)
**Census Region 3: Midwest**	2003	1.09 (1.01-1.17)
**Census Region 3: Midwest**	2004	1.01 (0.93-1.09)
**Census Region 3: Midwest**	2005	1.08 (1-1.15)
**Census Region 3: Midwest**	2006	1.06 (0.98-1.13)
**Census Region 3: Midwest**	2007	0.98 (0.91-1.06)
**Census Region 3: Midwest**	2008	0.98 (0.91-1.05)
**Census Region 3: Midwest**	2009	0.95 (0.88-1.02)
**Census Region 3: Midwest**	2010	0.9 (0.83-0.97)
**Census Region 3: Midwest**	2011	0.97 (0.9-1.04)
**Census Region 3: Midwest**	2012	0.97 (0.9-1.04)
**Census Region 3: Midwest**	2013	1.04 (0.97-1.12)
**Census Region 3: Midwest**	2014	0.99 (0.92-1.06)
**Census Region 3: Midwest**	2015	1.15 (1.08-1.23)
**Census Region 3: Midwest**	2016	1.1 (1.03-1.17)
**Census Region 3: Midwest**	2017	1.19 (1.11-1.26)
**Census Region 3: Midwest**	2018	1.19 (1.11-1.26)
**Census Region 3: Midwest**	2019	1.23 (1.16-1.31)
**Census Region 3: Midwest**	2020	1.59 (1.5-1.67)
**Census Region 4: Northeast**	1999	0.96 (0.88-1.05)
**Census Region 4: Northeast**	2000	0.97 (0.89-1.05)
**Census Region 4: Northeast**	2001	1.01 (0.93-1.09)
**Census Region 4: Northeast**	2002	1.02 (0.94-1.11)
**Census Region 4: Northeast**	2003	1.01 (0.93-1.1)
**Census Region 4: Northeast**	2004	1.01 (0.93-1.1)
**Census Region 4: Northeast**	2005	0.99 (0.91-1.07)
**Census Region 4: Northeast**	2006	0.88 (0.81-0.96)
**Census Region 4: Northeast**	2007	0.9 (0.82-0.97)
**Census Region 4: Northeast**	2008	0.87 (0.79-0.94)
**Census Region 4: Northeast**	2009	0.81 (0.74-0.88)
**Census Region 4: Northeast**	2010	0.89 (0.82-0.96)
**Census Region 4: Northeast**	2011	0.84 (0.76-0.91)
**Census Region 4: Northeast**	2012	0.88 (0.8-0.95)
**Census Region 4: Northeast**	2013	0.89 (0.82-0.96)
**Census Region 4: Northeast**	2014	0.88 (0.81-0.95)
**Census Region 4: Northeast**	2015	0.9 (0.83-0.97)
**Census Region 4: Northeast**	2016	0.93 (0.86-1)
**Census Region 4: Northeast**	2017	0.84 (0.78-0.91)
**Census Region 4: Northeast**	2018	0.84 (0.77-0.91)
**Census Region 4: Northeast**	2019	0.93 (0.86-1)
**Census Region 4: Northeast**	2020	1.13 (1.05-1.21)

Supplementary Table S7. Cirrhosis related mortality AAMR in patients with DM per 100,000 stratified by census region in the United States from 1999 to 2020

State	Age Adjusted Mortality Rate (95% CI)
**Alabama**	1.04 (0.98-1.1)
**Alaska**	0.75 (0.6-0.93)
**Arizona**	1.04 (0.99-1.09)
**Arkansas**	1.09 (1.01-1.16)
**California**	1.53 (1.5-1.56)
**Colorado**	1.12 (1.05-1.18)
**Connecticut**	0.82 (0.76-0.87)
**Delaware**	1.07 (0.93-1.2)
**District of Columbia**	0.79 (0.64-0.94)
**Florida**	0.81 (0.79-0.84)
**Georgia**	0.77 (0.73-0.81)
**Hawaii**	0.98 (0.87-1.08)
**Idaho**	1.04 (0.93-1.14)
**Illinois**	0.88 (0.85-0.92)
**Indiana**	1.26 (1.2-1.31)
**Iowa**	0.98 (0.91-1.05)
**Kansas**	0.96 (0.88-1.03)
**Kentucky**	1.97 (1.89-2.05)
**Louisiana**	0.85 (0.79-0.9)
**Maine**	1.01 (0.91-1.11)
**Maryland**	1.11 (1.05-1.17)
**Massachusetts**	0.84 (0.79-0.88)
**Michigan**	0.98 (0.94-1.01)
**Minnesota**	1.19 (1.13-1.25)
**Mississippi**	1.22 (1.14-1.3)
**Missouri**	0.93 (0.88-0.98)
**Montana**	0.84 (0.73-0.95)
**Nebraska**	0.94 (0.85-1.03)
**Nevada**	0.66 (0.59-0.72)
**New Hampshire**	1.1 (0.98-1.21)
**New Jersey**	0.87 (0.83-0.91)
**New Mexico**	1.82 (1.7-1.94)
**New York**	0.78 (0.75-0.81)
**North Carolina**	1.34 (1.29-1.39)
**North Dakota**	0.79 (0.65-0.92)

**Ohio**	1.46 (1.42-1.5)
**Oklahoma**	2.25 (2.16-2.35)
**Oregon**	1.35 (1.28-1.42)
**Pennsylvania**	1.15 (1.11-1.18)
**Rhode Island**	1.59 (1.44-1.74)
**South Carolina**	1.19 (1.13-1.26)
**South Dakota**	0.99 (0.86-1.13)
**Tennessee**	1.57 (1.51-1.63)
**Texas**	2.2 (2.15-2.24)
**Utah**	0.95 (0.86-1.04)
**Vermont**	1.45 (1.27-1.64)
**Virginia**	0.98 (0.93-1.02)
**Washington**	1.24 (1.19-1.3)
**West Virginia**	1.91 (1.79-2.03)
**Wisconsin**	0.93 (0.88-0.98)
**Wyoming**	0.98 (0.81-1.15)

Supplementary Table S8. Cirrhosis related mortality AAMR in patients with DM per 100,000 stratified by state in the United States from 1999 to 2020

Year	APC Values
**Overall**	
**1999-2003**	2.90 (0.82 to 8.72)
**2003-2009**	-1.98 (-5.72 to -0.54)
**2009-2018**	2.93 (1.63 to 4.24)
**2018-2020**	14.21 (9.33 to 17.29)
**Male**	
**1999-2001**	8.21 (2.57 to 13.53)
**2001-2010**	-0.69 (-2.97 to -0.07)
**2010-2018**	3.16 (1.82 to 4.37)
**2018-2020**	12.71 (8.34 to 15.43)
**Female**	
**1999-2003**	2.88 (0.73 to 8.37)
**2003-2009**	-2.16 (-5.77 to -0.79)
**2009-2018**	2.63 (1.36 to 3.83)
**2018-2020**	14.35 (8.00 to 18.18)
**Large Central Metropolitan regions**	
**1999-2003**	2.84 (0.57 to 9.47)
**2003-2009**	-2.01 (-5.81 to -0.45)
**2009-2018**	2.27 (0.82 to 3.92)
**2018-2020**	13.83 (8.06 to 17.30)
**Noncore (Nonmetropolitan) regions**	
**1999-2002**	7.03 (2.24 to 17.13)
**2002-2010**	-0.79 (-6.64 to 0.50)
**2010-2018**	5.93 (1.48 to 7.77)
**2018-2020**	14.51 (7.72 to 18.49)
**American Indian or Alaska Native**	
**1999-2013**	-2.59 (-5.93 to -0.12)
**2013-2020**	11.75 (7.43 to 20.54)
**Asian or Pacific Islander**	
**1999-2018**	-0.49 (-6.47 to 2.36)
**2018-2020**	15.66 (0.23 to 27.63)
**Black or African American**	
**1999-2018**	-0.37 (-1.68 to 0.22)
**2018-2020**	11.23 (0.73 to 16.59)

	**White**	
**1999-2003**		3.45 (1.31 to 9.17)
**2003-2009**		-1.87 (-5.72 to -0.48)
**2009-2018**		3.26 (1.98 to 4.51)
**2018-2020**		14.14 (9.28 to 17.20)
	**Hispanic or Latino**	
**1999-2018**		-0.17 (-1.14 to 0.51)
**2018-2020**		15.28 (3.64 to 20.92)
	**Census Region 1: Northeast**	
**1999-2018**		-0.93(-1.75 to -0.46
**2018-2020**		15.30(3.18 to 20.78)
	**Census Region 2: Midwest**	
**1999-2003**		3.59(0.29 to 14.19)
**2003-2010**		-2.32(-7.74 to -0.64)
**2010-2018**		3.10(-0.23 to 5.66)
**2018-2020**		14.26 (6.34 to 19.38)
	**Census Region 3: South**	
**1999-2003**		5.30(2.77to 11.82)
**2003-2007**		-3.32 (-6.63 to -0.36)
**2007-2018**		3.11 (2.16 to 4.25)
**2018-2020**		16.20 (9.54 to 19.79)
	**Census Region 4: West**	
**1999-2010**		-0.20 (-5.84 to 6.13)
**2010-2018**		2.90 (-2.54 to 4.90)
**2018-2020**		11.26 (4.17 to 15.89)
	**Age Groups**	
**1999-2001**	**25-44**	26.82 (0.16 to 59.48)
**2001-2012**	**25-44**	-5.64 (-18.48 to -3.29)
**2012-2020**		6.44 (1.505 to 21.27)
**1999-2018**	**45-64**	1.65 (-0.07 to 2.29)
**2018-2020**	**45-64**	12.34 (2.43 to 17.46)
**1999-2011**	**65+**	-0.48 (-1.44 0.11)
**2011-2018**	**65+**	4.12 (2.00 to 5.76)
**2018-2020**	**65+**	14.49(9.05 to 17.91)

Supplementary Table S9. Summary APCs of Cirrhosis-related AAMR in Patients with Diabetes Mellitus per 100,000 in the United States from 1999 to 2020
